# Developing an analytical method for quantification of trientine based on modified silver nanoparticles

**DOI:** 10.1186/s13065-023-01068-0

**Published:** 2023-11-08

**Authors:** Mahsa Khodadadi, Ali Shayanfar

**Affiliations:** 1 Student Research Committee, Faculty of Pharmacy, Tabriz University of Medical Sciences, Tabriz, Iran; 2 Biotechnology Research Center, Tabriz University of Medical Sciences, Tabriz, Iran; 3 Pharmaceutical Analysis Research Center, Tabriz University of Medical Sciences, Tabriz, Iran; 4 Faculty of Pharmacy, Tabriz University of Medical Sciences, Tabriz, Iran

**Keywords:** Plasma, Selectivity, Silver nanoparticles, Trientine

## Abstract

Trientine or (N,N´-bis(2-aminoethyl)-1,2-ethanediamine (TETA) is a copper chelator and used in Wilson’s disease, is aliphatic amine that does not have UV absorbing groups. In this study, the modified silver nanoparticles (AgNPs) by sodium lauryl sulfate have been used to develop an analytical method for quantification of TETA. Different concentrations of TETA were added into a particular concentration of AgNPs and absorbance of each sample was measured at 397 nm under the optimal conditions which include pH, time, salt and AgNPs volume. It was optimized by a design of experiments using response surface methodology. Then, the calibration curve was obtained based on the concentrations of TETA solution versus decrease in the absorbance of AgNPs. Selectivity of the developed method was performed in plasma and presence of common cations i.e. copper, zinc and ferrous. Under optimum conditions, linear range of this method was between 10 and 40 ng.mL^− 1^ with correlation coefficient (R^2^) of 0.996 with limit of detection and quantification of 3 ng.mL^− 1^ and 10 ng.mL^− 1^, respectively. Selectivity of established method in presence of cations eliminated by diluting because of high sensitivity of the established analytical techniques based on AgNPs. This method is suitable and low costing for quantification of TETA and does not require high equipment.

## Introduction

**Fig. 1:**
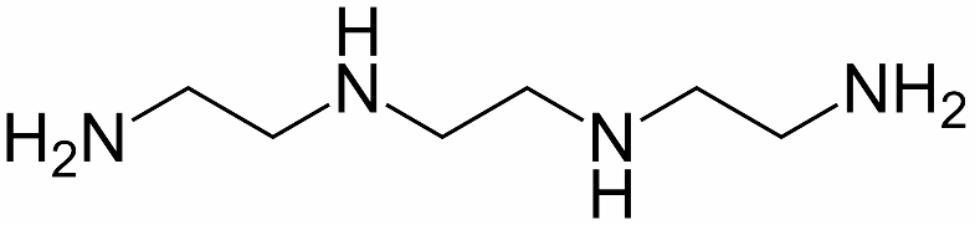
Trientine (triethylenetetramine, TETA)

Trientine with the chemical name triethylenetetramine (N,N´-bis(2-aminoethyl)-1,2-ethanediamine, TETA) (Fig. 1), is an amine compound that was presented as an alternative to penicillamine in severely suffering Wilson’s patients [1] with neurologic symptoms and in patients with hepatic failure [2, 3]. However, it has been used as initial therapy by showing efficacy in treatment of patients with severe liver or neurologic indications [4, 5]. Copper has coordinates bound by nitrogens in the TETA [6]. Studies have shown that copper chelators like TETA could re-sensitize cancer cells resistant to platinum by promoting the human copper transporter 1 (hCtr1)-mediated uptake of platinum [7]. In addition, this medicine can use in diabetes to reverse diabetic heart failure [8] and hypertrophic cardiomyopathy [9].

The measurement of aliphatic amines such as TETA have some problems e.g. low ultraviolet (UV) detection is due to the lack of UV absorbing groups [10]. This problem was solved by derivatization of them with other reagents. Bauer and Richter proposed forming the azomethine by pre-column derivatization of amine compounds with salicylaldehyde that were stable in basic solutions only. Time-consuming (16 h for each analysis) and high cost of the method are the main disadvantages [11].

Some methods have been proposed for analysis of TETA as an aliphatic amine. However, the most of them are complex analytical methods and have low sensitivity. Nakano et al. [12] reported a fluorescence derivatization method based on intramolecular excimer-forming. In this method, TETA and 1,6-hexanediamine (internal standard) with a pyrene reagent were transformed to the consistent excimer-forming derivatives i.e., (4-(1-pyrene)butyric acid N-hydroxysuccinimide ester). Miyazaki et al. [13] developed a determination method of TETA by the formation of fluorescamine derivatives. Another fluorometric method was proposed by Kodama et al. [14] to quantify TETA using HPLC system coupled with on-line post column derivatization. This method has lower sensitivity than the previous one, however, it does not need complex procedures to prepare samples. Hansen et al. [15] described a reversed-phase ion-pairing HPLC and conductivity detection method to determine TETA without need for derivatization reactions. They determined the stability of TETA during ambient storage and after autoclaving by performing nuclear magnetic resonance (NMR) spectroscopy. In 2007, Lu et al. [16], presented liquid chromatography–mass spectrometry method (LC–MS) to detect and quantify of TETA in plasma and urine samples which is time-consuming and an expensive technique for routine analysis.

A group of materials that have been investigated as colorimetric probes are metal nanoparticles [17–19]. These materials have a positive surface charge that can be changed their surface plasmon by adding substances with different characteristics and the tendency to bind to nanoparticles, and this feature can be used to analyze the medications [20–23]. Silver nanoparticles (AgNPs) are used as analytical and bioanalytical sensors caused of their distinctive visual, electrical, and chemical properties. Spectral properties of AgNPs are dependent on their shape, size, environment and the space between nanoparticles, hence, the geometry of AgNPs would control visual characteristics [24, 25]. They modified by sodium lauryl sulfate (AgNPs-SLS) due to having a negative charge on the surface of nanoparticles, can use for measuring substances that have amine groups [26]. The stability of these nanoparticles is because of the negative capping agent’s electrostatic repulsion in contrast to van der Waals attraction between AgNPs [27]. By attachment of different materials on the surface of AgNPs, surface plasmon resonance (SPR) band would be changed and various sizes of particles due to aggregation of AgNps causes converting the color from bright yellow to other colors [28–30]. These particulars can be used for analysis the materials.

Here, we report a cheap and rapid method for determining TETA by utilization of modified AgNPs to use UV-spectra detection that there is no need to large and expensive equipment.

## Materials and methods

### Materials

Silver nitrate, sodium lauryl sulfate, sodium borohydride, triethylene tetramine (TETA), zinc sulfate (ZnSO_4_.7H_2_O), ferrous sulfate (FeSO_4_.7H_2_O), copper sulfate (CuSO_4_), sodium hydroxide (NaOH), SLS and hydrochloric acid (HCl) was purchased from Merck (Darmstadt, Germany). Sodium chloride extra pure from Dr. Mojallali chemical laboratories (Tehran, Iran) was provided. Lab-made distilled water was used in all stages of experiments. pH was adjusted using a Metrohm Model 744 pH meter (Herisau, Switzerland) and A Shimadzu UV spectrophotometer (Analytik Jena AG, Germany) were used for UV measurements. Dynamic light scattering (DLS, Malvern Instruments, UK) was applied for determination of nanoparticle size.

### Synthesis and characterization of AgNPs

AgNPs was prepared by the method described in literature [26] with slight modifications. In this method, NaBH_4_ was used as reducing agent and SLS was used as stabilizer. Appropriate amounts of NaBH_4_ (38 mg) and SLS (85 mg) were dissolved in 50 mL distilled water and stirred at 380 rpm for 30 min. In another flask, 170 mg AgNO_3_ was dissolved in 50 mL distilled water. Then, AgNO_3_ solution added to the NaBH_4_-SLS solution dropwise and the final solution stirred for one hour. Prepared AgNPs-SLS was kept at room temperature (25°C) for one week to be ready for analysis. Finally, 1.5 ml of prepared nanoparticles solution was diluted in 50 mL volumetric flask with distilled water before analysis.

**Fig. 2:**
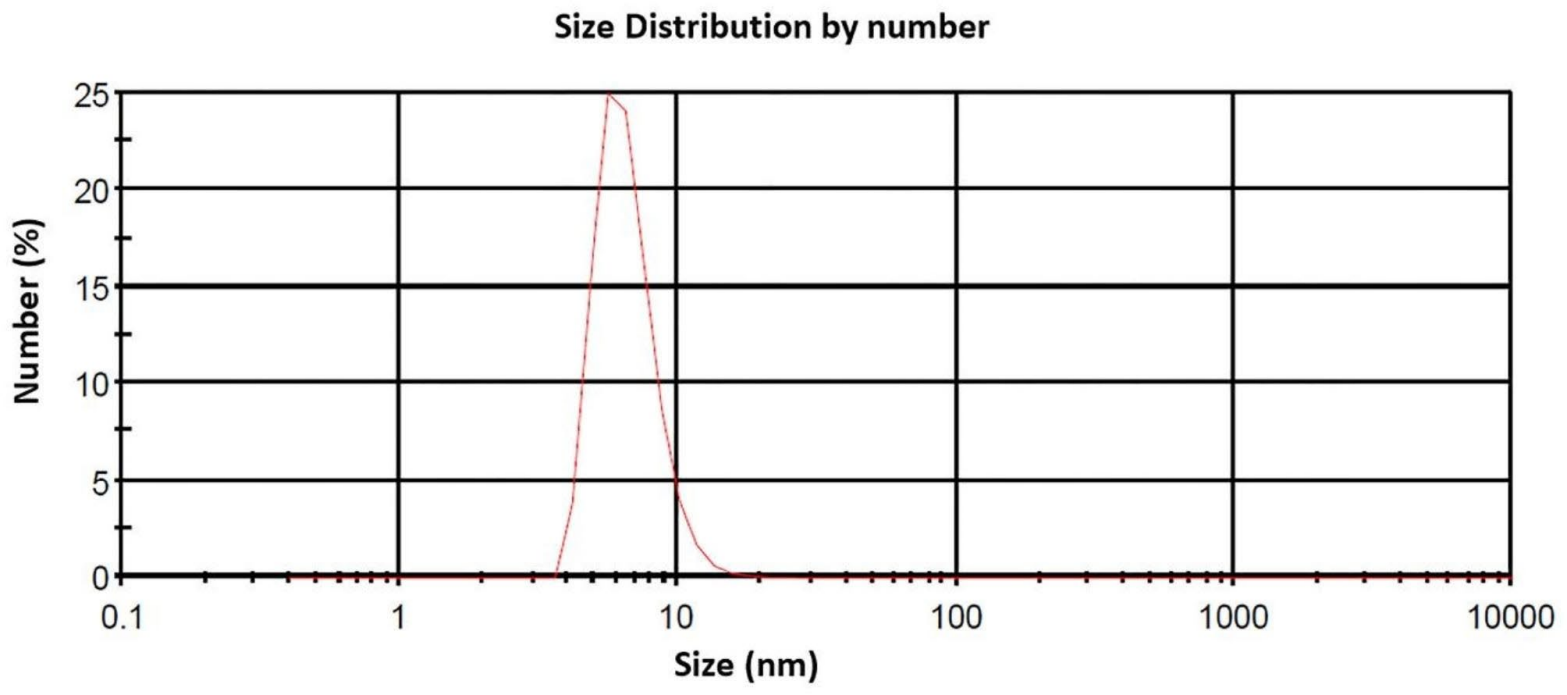
Dynamic light scattering (DLS) of the synthesized AgNPs

The synthesized AgNPs were characterized by DLS which shows nanoparticles size. The obtained DLS measurement of AgNPs is demonstrated in Fig. 2 indicates that the mean particle size of prepared AgNPs is 6.63 ± 1.82 nm.

### Experiment

Experimental design by response surface methodology (Box-Behnken) was performed by Minitab 17 software to optimize parameters. Based on preliminary experiments, different ranges of pH (2 [HCl 0.01 M], 7 (water), 12 [NaOH 0.01 M]), AgNPs volume (1-1.5 mL), NaCl volume (10–50 µl from 0.1 M solution) and time (5–15 min) were entered into software and 28 experiments was obtained to get the optimized conditions.

The optimized concentration of materials which includes 1.5 mL AgNPs, 10 µL NaCl from 0.1 M solution and 200 µL HCl from 1 M solution (final pH was 2) and different concentrations of TETA were added to series of 2 mL microtubes. Then, the mixtures were diluted up to 2 mL with distilled water. Absorbance of each sample was measured at 397 nm (maximum wavelength of absorption) and calibration curve (linear range between the concentration of TETA and decrease in the absorbance of AgNPs) was obtained. To check the accuracy and precision of method, inter-day and intraday was performed in three different concentrations of TETA.

### Selectivity

Selectivity of established method was checked in the presence of the biological ranges of common cations i.e. copper, zinc. Changes in absorbance of AgNPs at different concentration of TETA were recorded in various concentration studied cations to find limit of quantification. Dilution of samples was selected as a simple method (10, 100, 500 and 1000 fold dilution) to remove interferences i.e. cations.

### Application of the developed method to plasma samples

Drug-free plasma samples were provided from the Iranian Blood Transfusion Research Center (Tabriz, Iran) and frozen at -4 °C until analysis. Three plasma samples spiked with different concentrations of TETA (10, 20 and 30 ng.mL^− 1^). After 1000-fold dilution with water, the experiments were performed according to the optimal conditions and the acquired absorption of each experiment was recorded and compared with the absorbance in the aqueous samples.

## Results and discussion

**Fig. 3:**
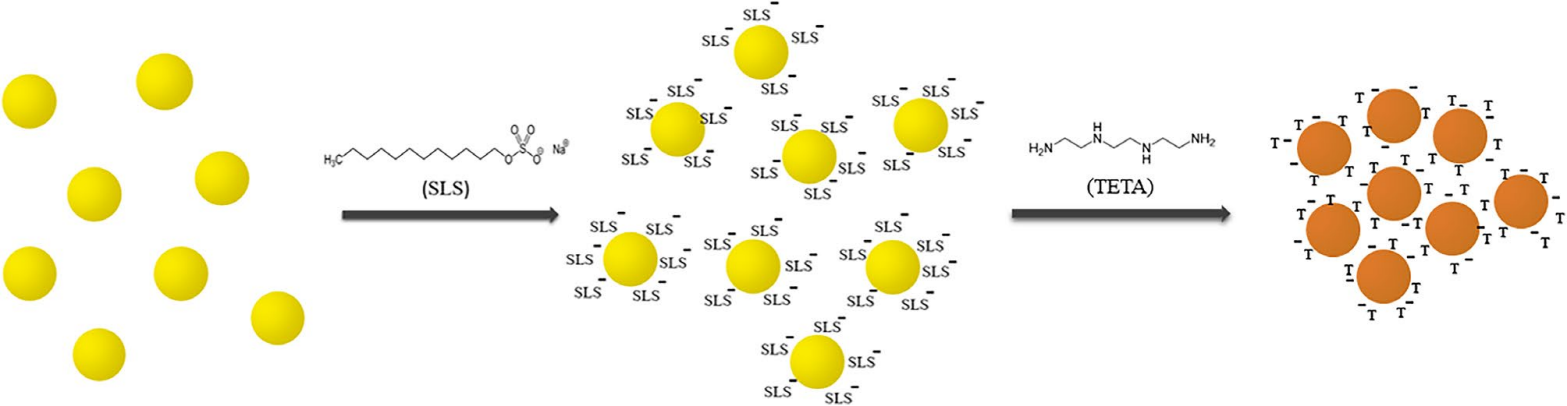
AgNPs-SLS aggregation and color changing by adding TETA

By adding analyte (TETA) to modified AgNPs, aggregation occurs and different colors depending on the concentration of TETA were observed (Fig. 3).

### Optimization of parameters

For optimization of developed method, experimental design by response surface methodology (Box-Behnken) was performed. Influential parameters in the testing process were entered into the software as independent variables and experiments were acquired. The difference in the absorbance of the samples and blanks (ΔAbsorbance) was considered in the response of each experiment. The experimental matrix and attained absorbance in each test have been reported in Table 1. From analysis of the results by stepwise multiple linear regression equation was obtained as following:

**Table 1:** Experimental design by response surface methodology to obtine optimized value of studied parameters

No.	pH	Time (min)	NaCl (µL)^a^	AgNPs (mL)	Δ Absorbance
1	7	10	10	1.00	0.58
2	7	10	30	1.25	0.92
3	7	5	30	1.50	0.98
4	2	10	30	1.50	1.26
5	2	15	30	1.25	-^b^
6	7	15	10	1.25	1.04
7	7	5	50	1.25	0.81
8	7	10	10	1.50	1.82
9	7	15	50	1.25	1.50
10	7	10	30	1.25	0.99
11	7	5	30	1.00	0.63
12	12	10	30	1.50	0.41
13	12	5	30	1.25	0.28
14	7	10	30	1.25	0.93
15	2	5	30	1.25	0.31
16	12	10	10	1.25	0.16
17	7	15	30	1.00	1.00
18	7	15	30	1.50	1.97
19	7	10	50	1.00	-^b^
20	2	10	30	1.00	0.76
21	2	10	50	1.25	0.92
22	12	10	50	1.25	0.06
23	12	10	30	1.00	0.23
24	2	10	10	1.25	1.22
25	7	5	10	1.25	0.49
26	7	10	50	1.50	0.57
27	12	15	30	1.25	0.40

^a^Stock solution (0.1 M)

^b^The 5th and 19th data have excluded because the analysis showed that these data caused error

ΔAbsorbance =–1.18 + 0.2575 pH – 0.0068 Time + 0.1306 NaCl – 1.98 AgNPs – 0.01542 pH× pH + 1.87 AgNPs× AgNPs − 0.01170 pH× Time + 0.1244 Time × AgNPs – 0.1045 NaCl × AgNPs.

**Fig. 4:**
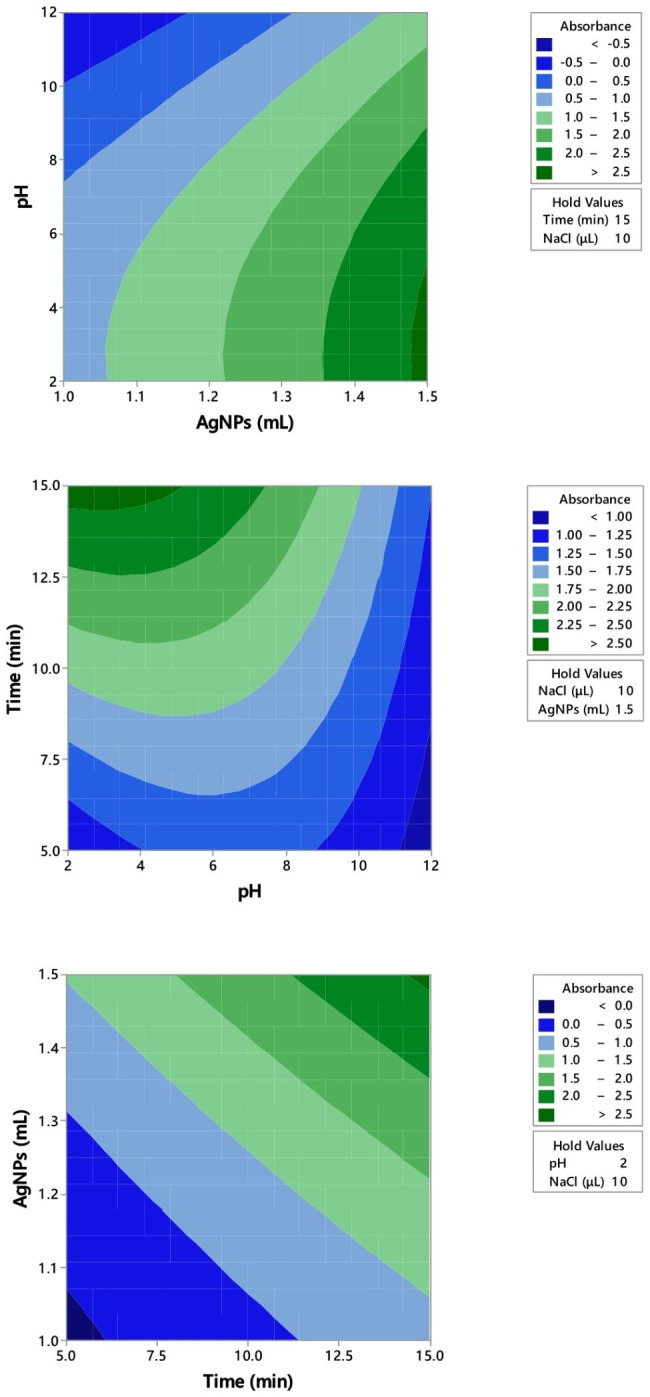
Contour plots of absorbance versus studied parameters (pH, time and AgNPs volume)

Correlation coefficient (R^2^), adjusted R^2^ and predicted R^2^ are 0.91, 0.86 and 0.71, respectively. P-value was gained from data analysis as reported in Table 2. P-value of all parameters was < 0.15 except for NaCl concentration. It indicates that the parameters and their interactions have statistically significant effect on absorbance. The data confirm the applied parameters have a noticeable impact on the absorbance except for NaCl concentration that has no significant effect (p-value = 0.98). In presence of NaCl, there was no noticeable increase in the critical coagulation concentration (CCC) for AgNPs [31]. However, its interaction parameter with AgNPs was substantial. Counter plots for pH, time and AgNPs concentration have been illustrated in Fig. 4. The results show that lower levels in pH and increasing the volume of AgNPs and time can decrease the absorbance of samples. SLS may increase particle stability by enhancing electrostatic and steric repulsion because of negatively charged molecules (SLS) and the compact coating layer, respectively [31]. At low pH, ionization of amine groups occur; that means there would be good attraction between TETA and SLS, so aggregation happens. On the way, negatively charged TETA is attracted by the positive charges on the surface of the AgNPs. Similar patterns have been reported for various drugs with amine functional groups [28–30]. Therefore, the optimum amounts of each parameter includes 15 mL of AgNPs, 10 µL NaCl, pH 2 and 15 min for time of reaction.

**Table 2:** Precision (inter-day and intra-day) and accuracy of the developed method

C _nominal_ (ng.mL^− 1^)	Average of absorbance	RSD%	C_observed_ (ng.mL^− 1^)	Accuracy %^a^
Intera-day				
15.0	1.69	9.7	16.1	107
25.0	1.43	6.3	22.8	91
35.0	1.04	11.1	32.6	93
**Inter-day**				
15.0	1.69	14.0	14.2	95
25.0	1.43	11.2	24.1	96
35.0	1.04	11.0	34.4	98

^a^(Obtained concentration/ Nominal concentration) × 100

### Calibration curve

**Fig. 5:**
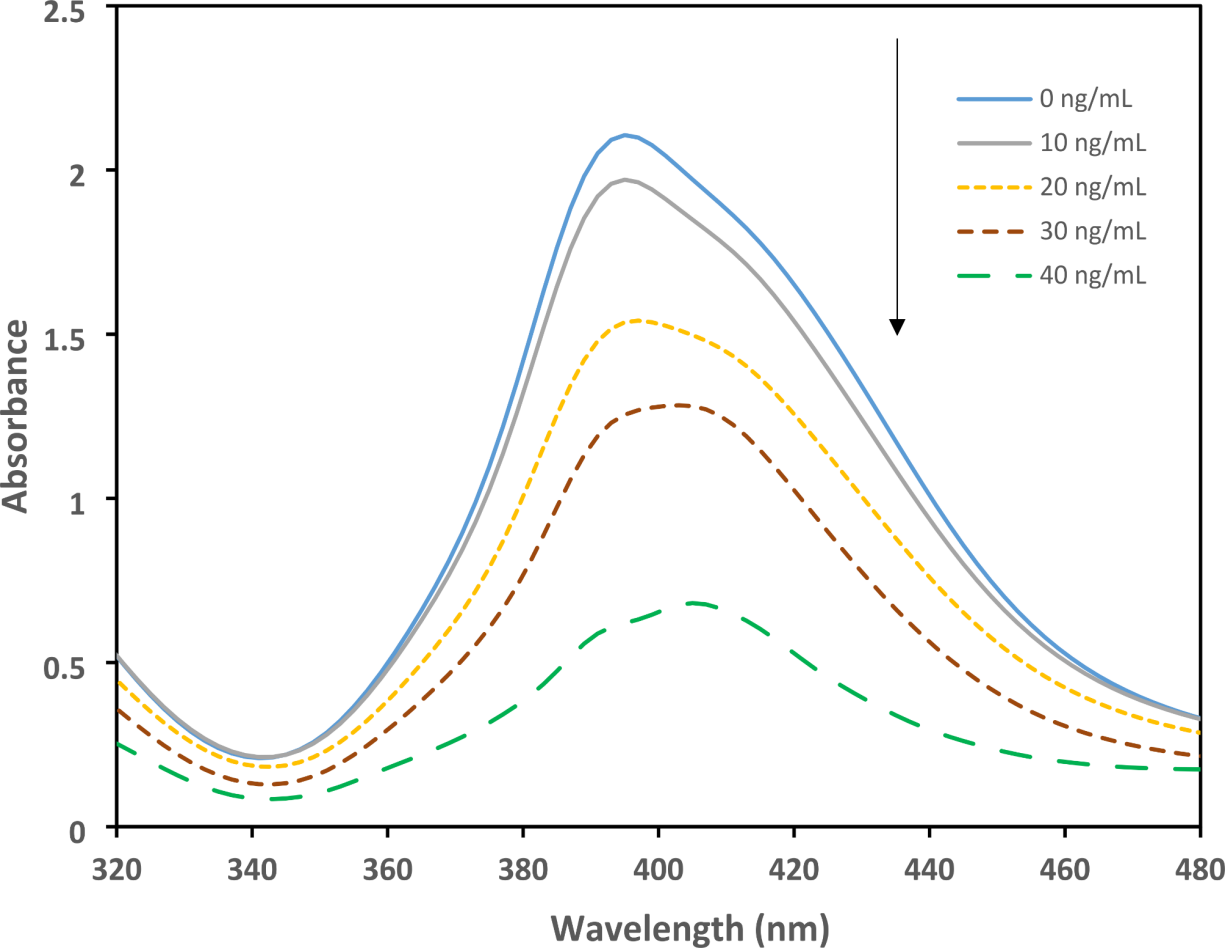
Effect of TETA (0–40 ng.mL^− 1^) on the UV-absorbance of AgNPs

The UV-visible spectrum of modified AgNPs solution have been illustrated in Fig. 5. It demonstrates that the UV-visible spectrum of AgNPs changes with increased concentrations of TETA, which indicates that the plasmon band of AgNPs is affected by the concentration of analyte. At lower concentration of TETA, the absorbance at 397 nm decreased slightly. By adding higher values of analyte, the absorbance band became weaker and broader; this reveals that interaction between TETA and AgNPs has formed.

According to experiments based on optimized variables, calibration curve was obtained by correlation coefficient (R^2^) of 0.996 that is linear between 10 and 40 ng.mL^− 1^ of TETA and following equation was acquired:

ΔAbsorbance=-39.06×Concentration + 2.32.

The limit of detection (LOD) and quantification (LOQ) is 3 ng.mL^− 1^and 10 ng.mL^− 1^, respectively. They were calculated by using standard deviation of blank (S_b_) and slope of calibration curve (m) as following:

LOD = 3(S_b_.m) and LOQ = 10(S_b_.m).

The coefficient of variation (CV) also known as relative standard deviation × 100 (RSD %) and relative recovery of back-calculated concentrations of calibration curve data points were less than 15% (except the last data which is less than 20%). These data, confirm that 10 ng.mL^− 1^, is lower limit of quantification (LLOQ) which is recommended parameter for evaluation sensitivity of method-based FDA guidelines for validation of small molecules [32].

### Accuracy and precision

To survey accuracy and precision, inter-day and intraday experiments were performed for three concentrations of analyte (15, 25 and 35 ng.mL^− 1^) two times a day for three different days. As reported data in Table 2, RSD% is less than 14%; therefore, precision of this method is within the acceptable range. By substituting the mean absorbance in obtained equation, the concentrations were calculated. The observed concentrations were within 91–107% of the nominal concentrations; so the accuracy for developed method is permissible. In conclusion, the results indicate that established method has acceptable accuracy and precision.

### Selectivity

**Fig. 6:**
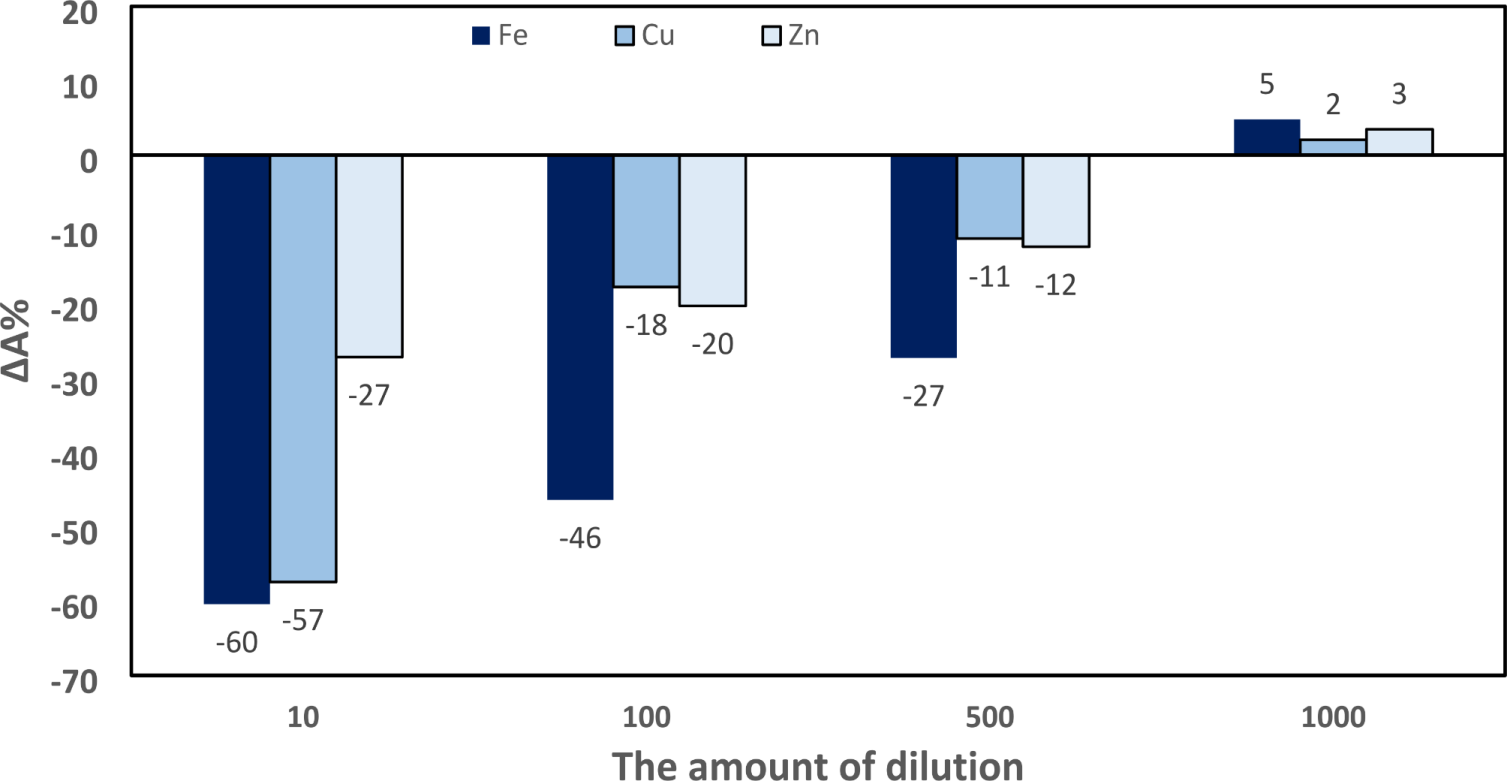
The changes in absorbance of AgNPs in presence ferrous (170 µg.dL^− 1^), copper (60 mg.dL^− 1^) and zinc (1 mg.dL^− 1^)

TETA is chelating agent of cations; therefore, it is necessary to check the selectivity in the presence of cations that present in blood i.e. copper that accumulates in Wilson’s disease, zinc which co-administrated with TETA in patients with Wilson’s disease and ferrous that is normally presents in blood and its supplements are routinely consumed. First, the changes in absorbance of the developed method were evaluated in presence of cations. Concentration of cations was selected based on their reported values in plasma i.e., ferrous 170 µg/dL [33], zinc 1 mg/dL [34] and copper 60 µg/dL [35] in Wilson’s patients. The results have depicted in Fig. 6. It indicates that after 1000-fold dilution of cation solutions, there is no significant difference in the absorbance of AgNPs in presence of 30 ng.mL^− 1^ of TETA, while 10, 100 and 500 times dilution have a considerable effect on the absorbance. These data confirm the importance of evaluating cations’ interference in developing analytical methods based on AgNPs. The high sensitivity of the established analytical techniques based on AgNPs and its administration with high dose allow that acceptable selectivity after 1000-fold dilution in the presence of cations and other drugs with similar structure i.e., structure with amine functional group such as beta blockers which has less plasma concentration than TETA.

### Application of method in plasma samples

To assess that developed method could be applied in biological samples, the experiments were performed on plasma samples. The difference in the absorbance of TETA in aqueous and plasma samples was in ± 15% after 1000-fold dilution. The plasma concentration of TETA in Wilson’s patients which administrated in high dose (> 1 g) and is approximately 1000 fold higher than the sensitivity of developed method [1]. Therefore, by diluting of the sample, TETA concentration would still be higher than the sensitivity of the established method.

### Comparison with other developed methods for quantification of TETA

Due to lack of UV absorbing groups in structure of TETA, it is hard to take UV spectrum; therefore, HPLC methods have been used for determination of amine compounds i.e. fluorescence, conductimetric and LC–MS methods. Conductivity detection was performed for analysis of TETA; however, limits of this method prevent application of that follow-up studies of these compounds in complex biological matrices. Flourometric methods using the pre-column derivatization of TETA are more sensitive but require elaborate preparation of the sample before analysis. HPLC-UV system coupled with post column derivatization of TETA, seems to be applicable to clinical use; however, sensitivity is lower than previous fluorometric methods. LC–MS method was developed to measure the TETA in plasma and urine which had proper sensitivity and can determine two metabolites of TETA but there some problems in developing the method to large-scale clinical analysis [13–16].

For determination of TETA, UV spectrum have not utilized because TETA does not have chromophore groups. We developed the method by AgNPs for analysis TETA in plasma which is facile method that there is no need for high equipment and analysis set up in 15 min against HPLC methods that are time-consuming and need high equipment. Comparison different methods based on chromatography techniques with various detectors in Table 3 indicates the high sensitivity of established method in this study. UV-spectrophotometry is simple and accessible instrumental analysis method which used for analysis of colorful samples in this study. Moreover, image analysis method e.g., RGB additive color model could be applied for developing a digital image colorimetric method for analysis of TETA. However, narrow linear range, the stability of AgNPs and selectivity of the method especially in the presence of cations and drugs with similar structure in complex matrices are limitations of the established method.

**Table 3:** Comparison with other developed methods for quantification of TETA

Method	Sample	Linear range	Ref.
HPLC-Fluorescence	Serum	0.15-15 µg/mL	[12]
HPLC-Fluorescence	Serum and urine	10–250 µg/mL	[14]
Reversed-phase ion-pairing HPLC-Conductivity	Aqueous solution	0.3–200 mg/mL	[15]
LC-MS	Plasma and urine	0.04–2.34 µg/mL	[16]
Spectrophotometry based on AgNPs	Aqueous solution and plasma*	0.01–0.04 µg/mL	This study

*After 1000-fold dilution with water, the method is selective in presence of the studied cations (ferrous, copper, and zinc) and could be applied for the quantification of TETA in plasma

## Conclusion

An analytical method based on AgNPs-SLS has been described for quantification of TETA which is a drug without chromophores by UV-spectrophotometry. The accuracy and precision of this method are acceptable and it has a short run-time against the other methods for quantification of TETA, which can be processed within 15 min. There are no need for complicated preparations i.e., extraction or precipitation of plasma proteins to put down interferences with the method. The interferences caused of proteins and cations in the plasma would eliminated by diluting because of high sensitivity of the established analytical techniques based on AgNPs. However, the selectivity of the method should be considered in analysis of complex matrices.

## Data Availability

Data generated or analyzed during this study are available from the corresponding author upon reasonable request.
